# On the representation and processing of social information in grounded cognitive systems: why terminology matters

**DOI:** 10.3389/fpsyg.2013.00180

**Published:** 2013-04-10

**Authors:** Kendall J. Eskine

**Affiliations:** Department of Psychological Sciences, Loyola University New OrleansNew Orleans, LA, USA

The mechanisms underlying social behavior are indeed complex, yet researchers have made important contributions to our understanding of how people make judgments and behave across various social contexts. In particular, recent years has seen a proliferation of research spotlighting the guiding role of embodied and affective information in social processing. Grounded approaches to cognition offer an exciting opportunity for researchers throughout the cognitive sciences to work within a unified framework to shed light on traditionally nebulous and intractable psychological quagmires (e.g., symbol grounding).

In what follows I will describe how embodied and affective information influence some hallmark social processes (moral judgments and prosociality) and then clarify some misunderstandings about representation and processing in grounded cognitive systems. I will then argue that the term “directionality” in grounded accounts engenders misleading views about cognition and will conclude with recommendations that should improve our understanding of social behavior from the growing perspective of grounded cognition.

A growing body of literature indicates that embodied and affective states influence moral judgments and prosociality. In the domain of moral judgment, research has shown that inducing physical disgust (via visual, olfactory, and gustatory senses) can harshen moral judgments (Schnall et al., 2008b; Eskine et al., 2011). The conceptual overlap between physical and moral disgust has been further confirmed with physiological evidence (Calder et al., 2001; Moll et al., 2005; Borg et al., 2008; Chapman et al., 2009). In a similar vein, researchers have also demonstrated that feeling physically clean and pure can license people to judge others morally harsher than those feeling dirty (Zhong et al., 2010). However, physical cleansing can also attenuate people's own moral guilt. Zhong and Liljenquist (2006) found that people felt less guilty about their own transgressions (and were more likely to volunteer) after they had cleansed themselves with an antiseptic wipe, whereas those who did not receive a wipe showed increased volunteerism (but see also Fayard et al., 2009).

This line of research is often described as a *moral purity* metaphor, in which physical purity is metaphorically projected onto conceptual representations of morality. However, the direction of these effects travels both ways. A disgusting taste in the mouth can harshen moral judgments (Eskine et al., 2011), but thinking about moral transgressions, virtues, or control events can lead people to perceive a neutral tasting beverage as disgusting, delicious, or neutral-tasting, respectively (Eskine et al., 2012; see also Ritter and Preston, 2011). Similarly, cleanliness can attenuate harsh moral judgments (Schnall et al., 2008a), while committing moral transgressions can enhance the desirability of cleansing products (Lee and Schwarz, 2010). The implications of directionality for grounded theories will be discussed.

In the domain of prosociality, Schnall et al. (2010) explored whether emotional elevation affected volunteerism (Study 1) and helping behavior (Study 2). Overall, they found that those who experienced elevation, but not other positive emotions like happiness or amusement, were more likely to volunteer for an unpaid study and help experimenters with a boring task compared to those in control conditions. Similarly, Liljenquist et al. (2010) tested whether clean scents affect financial decisions in an economic trust game with the prediction that clean scents will prime purity and thus enhance altruism. Results confirmed that those in clean-scented rooms gave more money to an alleged team-mate compared with those in baseline rooms. They replicated these findings by showing that clean-scented rooms also encouraged volunteerism and monetary donations for helpful causes.

To determine whether there is any psychological truth in common taste metaphors like “she/he's a sweetie,” Meier et al. (2012a) found that preferences for sweet foods significantly predicted prosocial behavior, and in another study they revealed that participants who consumed sweet foods (chocolate) were more likely to help another than those who consumed non-sweet foods (cracker) or nothing. These findings indicated that taste is an important embodied source domain that is projected onto abstract domains like prosociality, which bolsters a conceptual metaphor view that grounds abstract meaning in embodied source domains. But to what extent do views like these overlap in their theoretical assumptions with other views of grounded cognition (e.g., simulation theories)?

Broadly, principles of grounded cognition assert that sensorimotor and perceptual experiences are instrumental in the representation and processing of concepts. Simulation (Barsalou, 1999, 2008) and conceptual metaphor (Lakoff and Johnson, 1980, 1999) approaches represent the two dominant theories of conceptual grounding. Simulation models posit that conceptual processing recruits (roughly) the same perceptual states that were originally instantiated during one's initial embodied experiences, and metaphorical models contend that concrete, embodied experiences are projected onto abstract target domains. Although both views are “embodied,” it remains unclear whether the origin and organization of conceptual knowledge ultimately reside in simulation-based models rooted in perceptual simulation or conceptual metaphors as explained by cognitive linguistics. I view this point as important but somewhat tangential with respect to the current debates on the structure of conceptual knowledge. Metaphorical theories are traditionally argued to have a unidirectional structure (concrete-to-abstract effects), whereas simulation theories imply bidirectionality (concrete-to-abstract and abstract-to-concrete effects). These views are compatible to the extent that they both ground meaning in embodied/affective states, yet the issue of directionality has caused concern among many researchers (Landau et al., 2010; IJzerman and Koole, 2011; Lee and Schwarz, 2012; Slepian et al., 2012).

Lee and Schwarz (2012) maintain that the manner in which concepts are generally represented (representational structure) does not necessitate how concepts will be processed in real-time cognition. They argue that conceptual representations can have unidirectional structure but can *still* reveal bidirectional effects when processed online. While their insights are accurate, this seems to be a point that should not require defending if one considers the interaction between conceptual representation and processing. Representational structure and online processing are intricately interwoven, so much so, that teasing the two apart, particularly in terms of causality (directionality), can seem at times like more of an exercise for the armchair than the laboratory. While classic findings from cognitive science have helped refine our understanding of representation and processing (e.g., the rejection of traditionally accepted semantic network models à la Collins and Quillian, 1969, and Collins and Loftus, 1975, in lieu of more complex connectionist models à la McClelland, 2000), this representation-processing distinction is a core component of Barsalou's (1999) perceptual symbol systems (PSS).

Sensorimotor activity that naturally accompanies various perceptual states becomes incorporated into the representational and processing structure of concrete (e.g., cats) and abstract (e.g., generosity) category domains. These embodied perceptual states are stored in memory and (partially) reactivated in bottom-up format during later conceptual processing. For example, many early experiences of interpersonal warmth naturally co-occur with physical warmth, such as cradling infants. Hence, perceptual experiences involved with physical and interpersonal warmth become part of the same representational and processing structure, which is one way to explain the now-popular finding that experiencing physical warmth can promote interpersonal warmth toward a stranger (Williams and Bargh, 2008). Here, the representations themselves are the patterns of neural activity that span different regions of the brain, specifically perceptual and motor areas. While they may have some rough-and-ready structure, the task demands, social context, embodiment, top-down knowledge, affective states, etc. will re-construct representational-processing paths on a case-by-case basis. Thus, it should be no surprise that embodied states can affect abstract judgments and vice versa (implying bidirectional structure); they all participate in the same conceptual domain.

Therefore, while Lee and Schwarz (2012) are correct in arguing for bidirectionality in conceptual metaphors, a significant aspect of cognition is glossed over. I would assert that metaphorical knowledge is organized along lines of connectivity, not directionality. Both Lakoff and Johnson's (1999) and Leeand Schwarz's (2012) explanation of metaphor indicate that embodied and abstract domains are linked with each other and acquired through experiential coactivation, which is theoretically consistent with PSS. This truth in itself obviates the need for discussion of directionality because the manner in which these representations are activated (concrete-to-abstract or abstract-to-concrete) is simply a matter of the task-demands and context for a given embodied agent.

If this is indeed the case, then (1) why such emphasis on directionality and (2) what does this mean for social behavior? First, conceptual metaphor theory was born (in part) out of research in linguistics (Lakoff and Johnson, 1980), and directionality matters in linguistic metaphors. For example, calling a “butcher a surgeon” is very different than calling a “surgeon a butcher.” The direction of the metaphor completely changes its meaning. Thus, directionality seems to be crucial to understanding/inferring meaning in linguistic metaphors, but it can be argued to be irrelevant to conceptual metaphors because brains do not process information in terms of rigid directionality; their processing is often determined by context-sensitive experiences that provide coactivations between various processing regions. Second, in addition to propagating misleading views about conceptual processing, another danger of overemphasizing directionality in grounded theories is that it can lead researchers down garden path research programs. Bidirectional and unidirectional effects can still be accommodated by simulation models, and both distract researchers from testing more specific models of groundedness. Third, though it is well-documented that metaphorical approaches can transfer embodied source domains to various dissimilar abstract/target domains (Landau et al., 2010), which implies directionality, context-sensitivity can still account for these differences, and it cannot be ruled out that more complex activation patterns in association areas of the brain undergird these effects. Therefore, since both PSS and conceptual metaphors appear to equally rely on such coactivations to create substrates for conceptual development, the use of the term “directionality” engenders misleading views about conceptual representation-processing and needlessly creates theoretical divisions among similar-minded researchers.

This clarification is particularly important for social behavior because conceptual knowledge has been demonstrated to be an important but *flexible* foundation that shapes people's social processing (see Lee and Schwarz, 2011, for evidence for context specificity). Therefore, I propose that we shift the language from “directionality” to “connectivity” to highlight the relative flexibility of conceptual systems, and how variability (culturally, contextually, and individually) helps determine how people think, judge, and act toward others. This view is also consistent with alternative metaphorical models that rely on “blends” to engender the kinds of temporary, embodied, contextually sensitive, and dynamical conditions that seems most consistent with what is currently known about cognitive processing (see Fauconnier and Turner, 1998, for a blending theory of metaphor that is more compatible with Lakoff and Johnson, 1999, than Lakoff and Johnson, 1980). Thus, while directionality is an appropriate tool for linguistic metaphors, it proves problematic for conceptual processing. Since these are separate domains, this proposed distinction will similarly be unable to accommodate linguistic metaphors because it is tooled for investigating conceptual processing.

In short, embodied/affective states not only help ground meaning and guide social processing but are also intricately linked to online processing and experience, upon which representational-processing states are founded. Along these lines, researchers have rightly argued that more attention should be given to cultural differences in metaphorical and embodied cognition (Meier et al., 2012b), which is better accommodated by “connective” rather than “directional” terminology, as the latter implies a certain amount of rigidity that fails to empirically occur in the brain or in social conceptual processing. In this way, there is considerable overlap in simulation and metaphorical views of grounded cognition.

Embodied effects that were once striking, intriguing, and perhaps confounding, are now commonplace, and it is indeed time to breathe life into these effects with systematic theory-building that is predicated on a deeper analysis of the mechanisms underlying grounded theories of cognition. By nature, embodied and affective information are flexible sources of information, which is evidenced by their context dependence (Lee and Schwarz, 2011), and researchers have just begun tapping into their malleable (and adaptive) properties. To better investigate the nuances of grounded theories of social behavior, I have proposed that we reconsider our terminology in simulation- and metaphorical-based approaches. Rather than focusing on aspects of directionality that are couched in linguistic theories, highlighting the connective properties that develop through coactivation seems like a more promising path that better reflects how the brain actually processes information.
